# Position-Specific Differences in Physical Fitness Characteristics Among Adult Male Rugby Players—Systematic Review

**DOI:** 10.3390/jfmk11030313

**Published:** 2026-08-11

**Authors:** Karol Czyż, Krzysztof Kasicki, Łukasz Rydzik, Winicjusz Sokół, Julita Kalinowska, Andrzej Kędra, Jarosław Jaszczur-Nowicki

**Affiliations:** 1 Ogniwo Sopot, “Ogniwo” City Sports Club, 81-741 Sopot, Poland; karolczyz@op.pl; 2 Department of Sport Theory and Motor Skills, Institute of Sports Sciences, University of Physical Culture in Kraków, 31-571 Krakow, Poland; 3 Foundation Omnia Pro Polonia, 02-591 Warsaw, Poland; wineks@poczta.onet.pl; 4 Department Tourism and Recreation, University of Warmia and Mazury, 10-719 Olsztyn, Poland; julita.kalinowska@uwm.edu.pl; 5 Department of Sport Science, Faculty of Social Sciences, Vincent Pol University, 20-853 Lublin, Poland; aakedra@poczta.onet.pl; 6 Department Physiotherapy, School of Public Health, Collegium Medicum, University of Warmia and Mazury, 10-719 Olsztyn, Poland; j.jaszczur-nowicki@uwm.edu.pl

**Keywords:** team sports, motor skills, body composition, match demands, athletic testing, physiological profile, performance

## Abstract

**Background**: Rugby union and rugby league are among the most physically demanding contact team sports, in which playing positions impose distinct physical requirements. The aim of the review was to collect and synthesize available data on positional differences in physical fitness between forwards and backs in adult male rugby players. **Methods**: The systematic review was conducted in accordance with PRISMA 2020 and SWiM guidelines and was prospectively registered in the OSF database (10.17605/OSF.IO/E4Y37). Four electronic databases were searched: PubMed, SPORTDiscus, Web of Science, and Scopus up to June 2026. The search identified 3058 records, which were reduced to 1343 after language and document-type limits and then screened. Eligibility criteria were applied within the PICOs framework. Methodological quality was assessed using JBI Critical Appraisal Checklists and the AXIS tool as a validation instrument. Due to considerable methodological heterogeneity, narrative synthesis with directional meta-synthesis was applied. **Results**: Thirty-seven studies were included (n > 2000 players; 15 countries; 1996–2025). Forwards achieved higher values of absolute muscular strength and power, body mass, and sprint momentum, whereas backs performed better in sprint tests (10–50 m), maximum sprint velocity, aerobic fitness (VO_2_max, Yo-Yo IRTL1), CMJ height, and relative power. Following normalization to body mass, positional differences in strength and power were attenuated or reversed. In the only study that isolated the change in direction deficit, no clear positional difference was observed. **Conclusions**: Body mass appears to be the principal explanatory factor underlying positional differences in absolute strength and power. Position-specific fitness profiles are observable as early as the U20 category and become more pronounced with increasing competitive level. Strength and conditioning coaches should compare positional groups using body mass normalized ratio or, where justified, allometrically scaled measures rather than absolute values.

## 1. Introduction

Rugby union and rugby league are among the most physically demanding contact team sports, characterized by a complex combination of high-intensity interval efforts, explosive actions, and repeated high-force collisions [1,2,3]. A single 80 min match involves hundreds of distinct actions, including sprints, tackles, rucks, mauls, lineouts, and scrums. This places demands on the ability to generate maximum muscle force, power, sprint speed, and cardiorespiratory capacity. These requirements distinguish rugby from most other team games and make conducting research on the physical fitness of players both scientifically difficult and crucial from the perspective of training practice.

A structural feature of both varieties of rugby is the differentiation of on-field roles, leading to a clear division of players into the forwards formation (FWD) and the backs formation (BL), with additional, more specific positional divisions used depending on the game code. In rugby union (15-a-side), forwards play in positions 1–8 and include props, hooker, second row, flankers, and number eight, while backs (positions 9–15) are the scrum-half, fly-half, centers, wings, and fullback. In rugby league (13-a-side), the analogous division most often distinguishes “hitup forwards,” “adjustables,” and “outside backs.” [4].

Forwards are primarily responsible for actions in set-piece phases such as scrums, lineouts, restarts, and for contact-oriented play, including taking the ball into the opponent’s defense, tackles, rucks, and mauls [1,3]. These tasks require the ability to generate high ground reaction forces, tolerate repeated high-force collisions, and develop maximum muscle strength and power over very short distances and in short time frames. Consequently, forwards are generally characterized by greater body mass and higher absolute muscle strength compared to backs. The largest body mass differences are noted at the elite and international level, where forwards outweigh backs by an average of 20–25 kg (ref. [1]: 116.5 vs. 95.9 kg [1]; ES = 2.10; ref. [2]: 108.3 vs. 84.0 kg; ES = 2.27; ref. [3]: 115.8 vs. 94.5 kg [3]), while at the amateur and sub-elite level, differences are usually 10–15 kg.

Backs, in turn, are responsible for distributing the ball in open play, conducting fast offensive and defensive actions across the full width of the pitch, and making quick decisions under physical pressure [5]. These requirements are associated with the necessity of achieving higher running speed, greater aerobic capacity ensuring the maintenance of high-intensity running for a longer period, and a high level of reactive agility. Data from GPS systems monitoring match loads show that backs cover a greater distance in high-intensity zones during a match and reach higher maximum sprint speeds than forwards. These results are confirmed by kinematic analyses by ref. [5], which showed that during the linear acceleration phase of a sprint, backs are characterized by shorter foot-ground contact time and higher step frequency, approaching track sprinters in terms of their mechanical profile, whereas forwards generate impulse mainly through longer contact with the ground, which is more consistent with the requirements of positions associated with physical contact during the game.

Taking into account the differentiation of positional requirements, the hypothesis that playing position constitutes an independent determinant of the physical fitness characteristics of rugby players has interested researchers since the early 1990s. Individual studies have usually analyzed single fitness domains, including: muscle strength, running speed, jump ability, aerobic capacity, agility, or repeated sprint ability. Despite this, the number of scientific publications on the subject has grown in a slow and episodic manner, meaning that at the present moment there is a lack of a comprehensive study discussing the basic differences in player performance relative to their position on the pitch.

Many unresolved issues remain in the literature. It is still unclear to what extent body mass explains the reported advantage of forwards in terms of strength and power. Additionally, the consistency of positional differences depending on the sporting level has not yet been analyzed; available evidence suggests that physical differences are smaller at the amateur and junior level than in professional and international populations, but this has not been formally classified. The distinction between change-of-direction speed and change-of-direction deficit has so far been examined in only one study [2], which constitutes a methodological gap in the area of agility research.

To date, few systematic reviews have simultaneously synthesized the available evidence regarding all key domains of physical fitness of rugby players, including, among others, muscle strength, muscle power, or aerobic capacity, while at the same time taking into account the variable impact of normalization relative to body mass, competition level, and rugby code. Moreover, existing reviews have generally focused on a single fitness domain, on GPS-derived match running demands, or on a single code or competition level, and none has systematically integrated forwards-versus-backs comparisons across all major fitness domains while explicitly quantifying the confounding contribution of body mass [3]; the present review was designed to address this gap.

The aim of this review was therefore:

(1) to collect and synthesize available data regarding positional differences in physical fitness and exercise characteristics between forwards and backs in adult male rugby populations, across all major physical fitness domains;

(2) to evaluate the extent to which body mass explains, as a scaling factor, the observed positional differences;

(3) to examine the consistency of positional differences depending on the competition level and game code (rugby union and rugby league);

(4) to assess the methodological quality of the included studies using validated assessment tools;

(5) to identify gaps in empirical literature and formulate recommendations for future research and coaching practice.

Of these aims, objectives (1) and (2) constitute the two central questions of the review—whether forwards and backs differ consistently across the key physical-fitness domains, and whether such differences are attenuated or reversed once outcomes are expressed relative to body mass. Objective (3), concerning competition level and rugby code, is treated as an exploratory subgroup consideration rather than a primary analytical aim, whereas objectives (4) and (5) represent standard reporting components of a systematic review. No formal test of statistical mediation was undertaken; accordingly, body mass is described throughout as an explanatory (scaling) variable and not as a demonstrated mediator.

## 2. Materials and Methods

This review was conducted in accordance with the SWiM [6] (Synthesis Without Meta-analysis in systematic reviews) guidelines and the PRISMA guidelines [7]. It was registered a priori in the OSF database under the registration number: 10.17605/OSF.IO/E4Y37.

### 2.1. Literature Search Strategy

**Figure 1:**
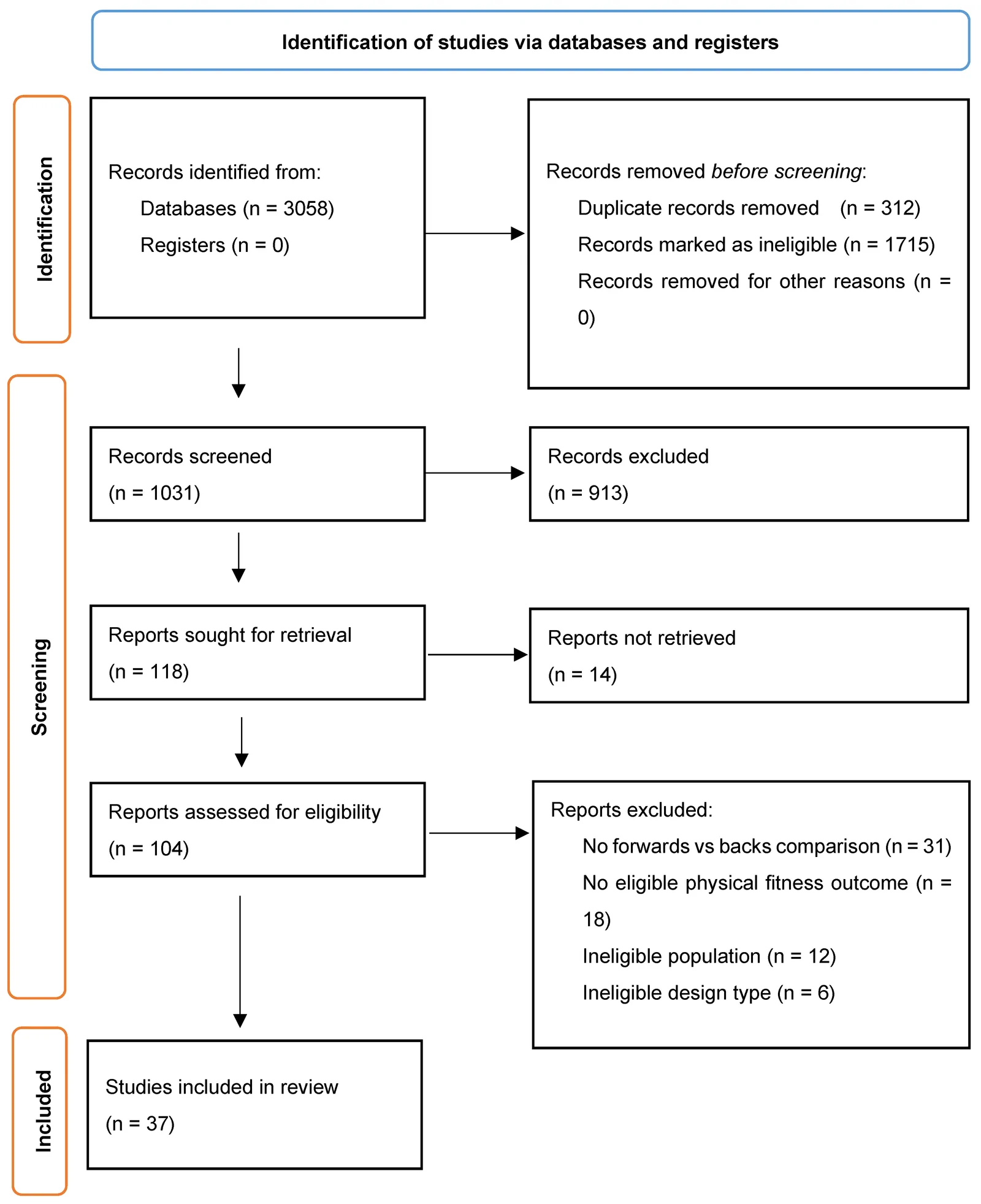
PRISMA 2020 flowchart.

In January 2026, one of the authors (K.K.) conducted an initial search in the following databases: PubMed, Scopus, Web of Science, and SPORTDiscus, covering all publications issued up to the end of January 2026. Based on the search phrases provided below for each database, 3058 results were initially identified. Subsequently, for each database, the following criteria were applied, which every result had to meet: English language and indexing as an original article in a peer-reviewed journal. No open-access or free-full-text limit was applied at this stage; where a full text could not be obtained, the record was subsequently logged as a report not retrieved in the study selection flow (Figure 1). After applying the filters, 1343 results were finally obtained. The results for each database were as follows: Scopus: 447; Web of Science: 491; SPORTDiscus: 374; and PubMed: 31. Detailed search phrases for each database are presented in Appendix A. The database search was updated in June 2026, immediately before manuscript submission; this update did not identify any additional studies meeting the eligibility criteria, so the final search date is reported as June 2026.

### 2.2. Eligibility Criteria

Based on the initial research concept, inclusion and exclusion criteria were established using the PICO model. Research on rugby players over the age of 18 was included at any sporting level, starting from amateur. Publications on women were not included due to the very small number of articles compared to studies conducted on men in this field of sport. Only English-language articles were selected; open-access status or free full-text availability was not used as an eligibility criterion. To keep the synthesis aligned with the stated adult-male scope, only data from samples with a mean age of at least 18 years contributed to the primary adult synthesis. A small number of studies additionally reported sub-adult data; these were retained solely within a clearly labeled developmental-context subsection and were not used to support any conclusion about adult players. Statements regarding the early emergence of position-specific profiles should therefore be read as developmental context requiring confirmation in dedicated youth studies, rather than as adult findings.

Specific types of studies, such as meta-analyses, reviews, and abstracts, were excluded. Detailed inclusion and exclusion criteria are presented in Table 1.

### 2.3. Selection Process and Data Management

All retrieved records obtained from the databases were imported into separate Excel files. Due to the small number of results obtained, the files containing the records for screening were divided among 3 authors. Each author (Ł.R., K.C., K.K.) individually and manually analyzed each result in accordance with the established inclusion and exclusion criteria. When one of the authors had difficulty determining the eligibility of a specific study, another independent author who was not performing the screening (J.J-N.) made the decision after a discussion with all team members. Finally, from each database and assigned file of results, a specific number of publications meeting the criteria was successfully selected. The last step was to combine the obtained results into a single file and perform manual deduplication by one of the authors (K.C.). Because records were divided among reviewers rather than each record being screened independently in duplicate by two reviewers, we acknowledge this as a deviation from fully independent duplicate screening; a second reviewer (J.J.-N.) adjudicated all uncertain cases, and title/abstract and full-text stages were cross-checked. The review was prospectively registered in OSF (https://doi.org/10.17605/OSF.IO/E4Y37); no deviations from the registered protocol occurred other than those reported here.

### 2.4. Data Collection Process

Two authors (K.C. and K.K.) performed the data extraction from the selected publications. The process was carried out manually for each publication included in this process. In the event of finding non-compliance with the inclusion or exclusion criteria, a decision was made to remove the publication from the list of those included in the review. Additionally, in case of a lack of consensus regarding data extraction, another independent author (Ł.R.) resolved the matter during a discussion with the entire team of authors regarding the correctness of the data extraction. Finally, a re-analysis of the data was introduced to assess its correctness, followed by the approval of all authors of the study.

### 2.5. Data Items

For each publication, the following groups of variables were extracted:

- Study characteristics: bibliographic data, study design, rugby type (i.e., rugby league or rugby union), level of competition
- Participants’ characteristics: sample size, age, positional classification divided into forwards and backs, anthropometric data
- Motor abilities and physical fitness: muscle strength, muscle power, sprint speed, aerobic capacity, agility and change in direction, as well as anaerobic capacity and repeated sprint ability
- Quantitative data: mean and standard deviation, and if available, 95% confidence intervals, *p*-value, effect size, statistical test, direction of difference: FWD > BL, BL > FWD or NS (non-significant). In the case of studies applying multi-position classifications, data for all sub-positions along with post hoc analyses.

To keep the scope internally consistent, extracted variables were assigned to three mutually exclusive categories.

1. Physical fitness tests were laboratory- or field-based assessments of motor capacity (muscle strength, muscle power, sprint speed, aerobic or endurance capacity, change-of-direction or agility, repeated-sprint ability, and anaerobic capacity) and constituted the outcomes of the review.
2. Anthropometric and contextual variables, for example, body mass, body height, and body composition, were extracted only as explanatory or scaling covariates and were not, on their own, treated as fitness outcomes.
3. Match-derived performance variables, for example, sprint momentum and match-derived maximum sprint speed, were reported separately and were not combined with standardized fitness-test outcomes.

### 2.6. Study Risk of Bias Assessment

**Figure 2:**
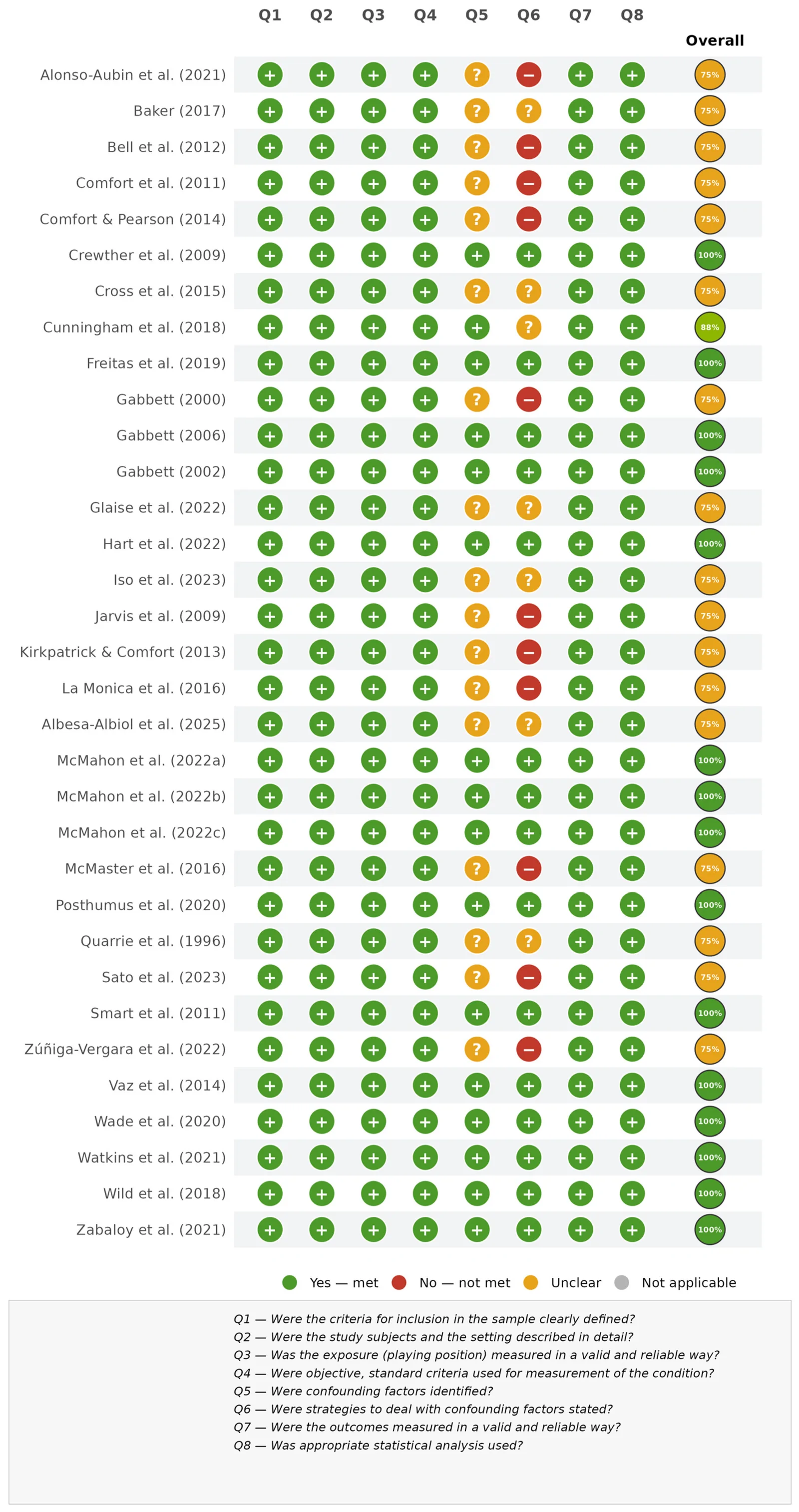
Traffic light plot JBI critical appraisal checklist for analytical cross-sectional studies (n = 33) [1,2,3,5,11,12,13,14,15,16,17,18,19,20,21,22,23,24,25,26,27,28,29,30,31,32,33,34,35,36,37,38,39].

**Figure 3:**
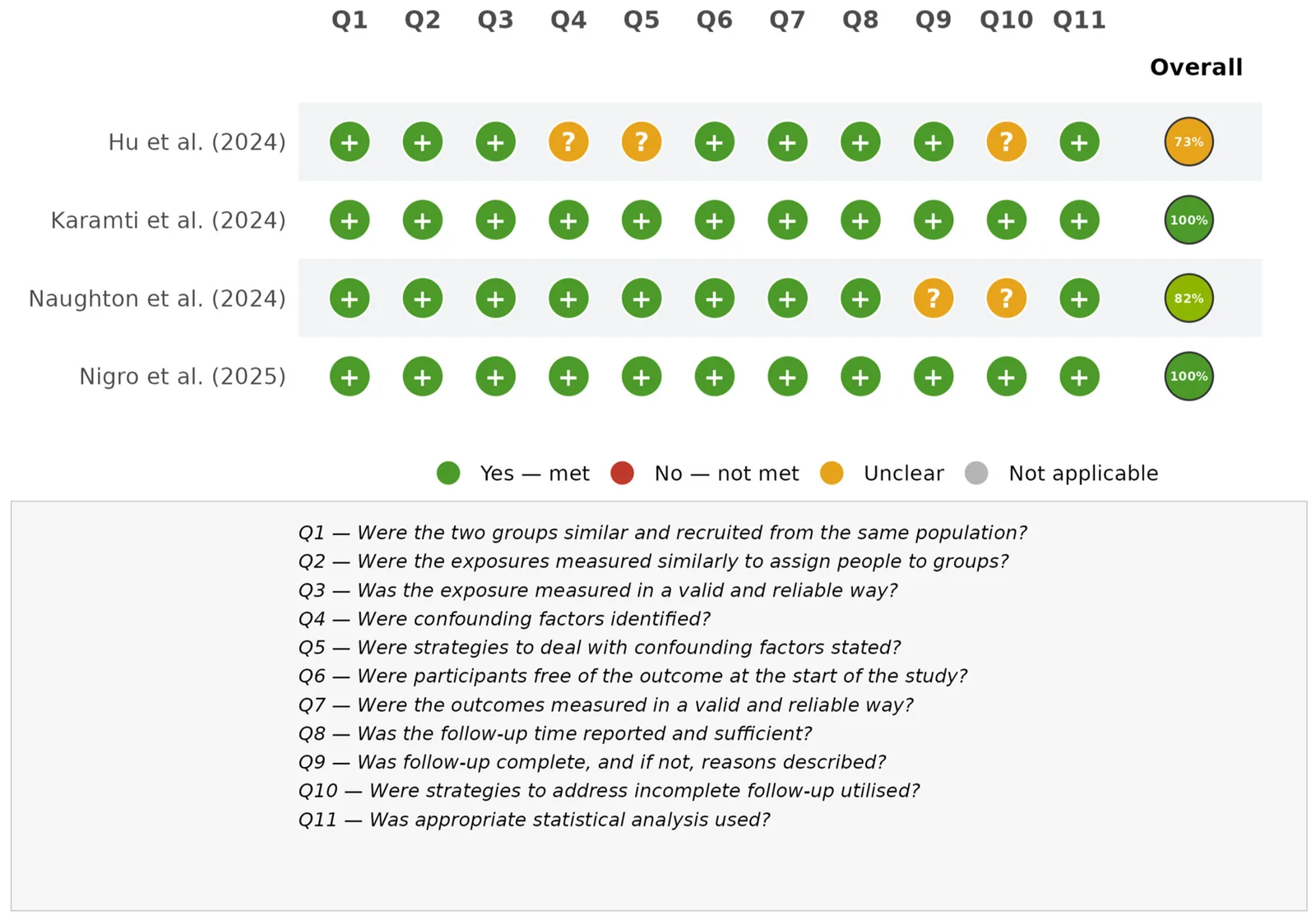
Traffic light plot—JBI critical appraisal checklist for cohort studies (n = 4) [40,41,42,43].

**Figure 4:**
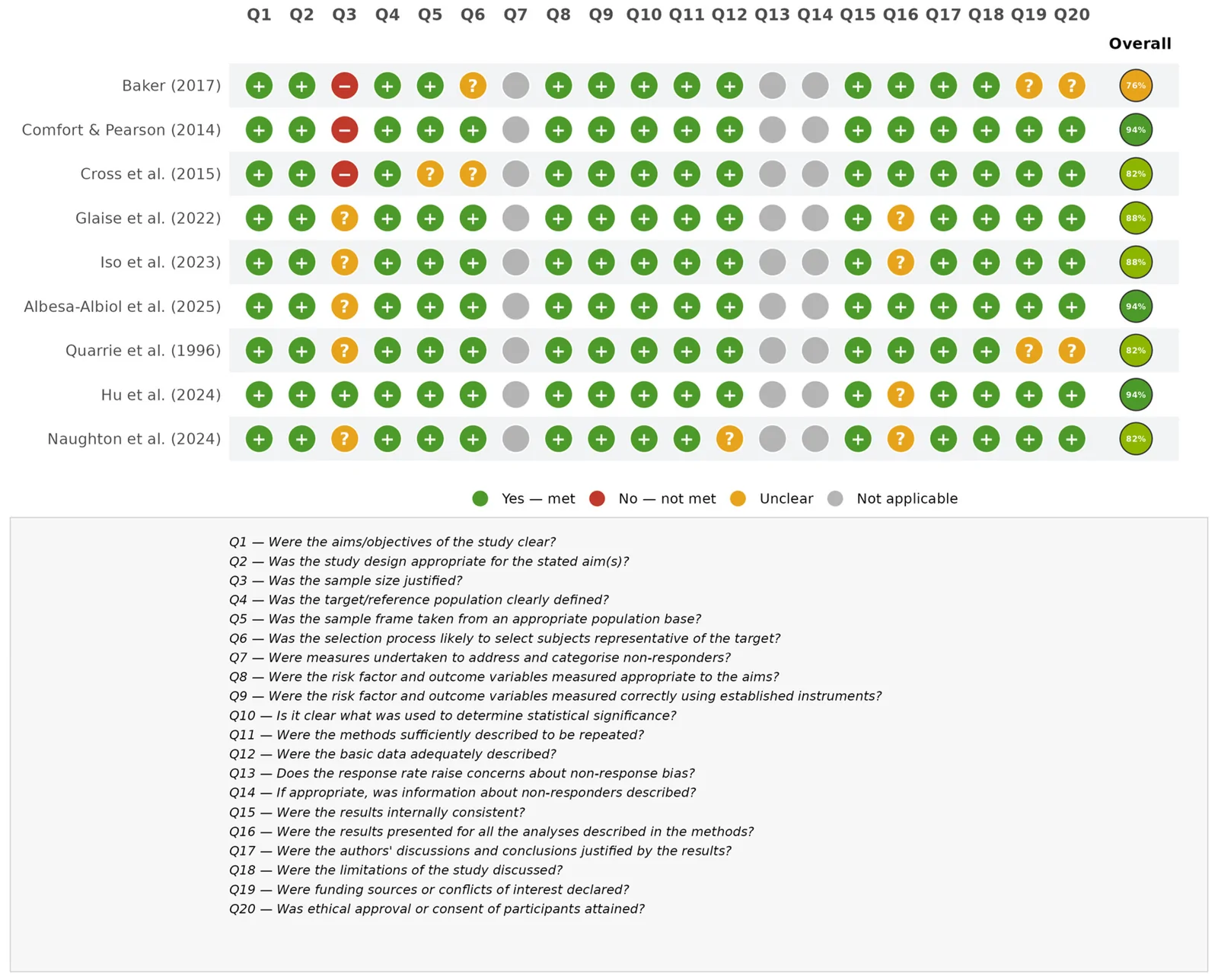
Traffic light plot AXIS appraisal tool—validation for studies with ambiguous JBI ratings (n = 9) [11,13,18,20,30,32,37,40,41].

The risk-of-bias appraisal was performed independently by two reviewers (K.C. and K.K.); disagreements were resolved by discussion and, where consensus could not be reached, adjudicated by a third author (Ł.R.). Because the JBI checklists yield categorical, item-level judgements rather than a validated summary score, the percentage of affirmative responses is reported only as a descriptive summary to aid cross-study comparison, and not as a validated quality scale; item-level traffic-light plots (Figure 2, Figure 3 and Figure 4) are provided so that individual domains can be inspected directly. The AXIS tool was applied as a sensitivity check for the subset of cross-sectional studies whose JBI confounding items were rated “Unclear”, in order to confirm that an ambiguous rating on a single domain did not misclassify overall study quality; AXIS was not used to compute an independent composite score.

To assess the risk of bias in the included studies, two scales were used. The first was the JBI Critical Appraisal Checklists developed by the Joanna Briggs Institute, and the second was AXIS—Appraisal Tool for Cross-Sectional Studies. The choice of these tools resulted from the specific nature of the included studies; that is, all 37 included papers were observational in nature: 33 cross-sectional and 4 longitudinal, which precluded the use of tools dedicated to randomized trials.

#### 2.6.1. JBI Critical Appraisal Checklist

For the 33 studies with a cross-sectional design, the JBI Critical Appraisal Checklist for Analytical Cross-Sectional Studies [8] was applied, which was adapted for the purposes of this work, encompassing eight evaluation questions: (1) clarity of defining the sample inclusion criteria; (2) detail of the description of the population and study settings; (3) reliability of exposure measurement: in this review, the playing position; (4) use of objective, standardized criteria for baseline assessment; (5) identification of potential confounding factors, such as age, competition level, training experience, or phase of the season; (6) applied strategies to control these factors; (7) reliability of measuring performance outcomes (measurement method, protocol standardization, equipment calibration, ICC values); and (8) adequacy of the applied statistical analysis.

For the 4 studies with a longitudinal design the JBI Critical Appraisal Checklist for Cohort Studies [9] was used, encompassing 11 evaluation questions assessing additionally: comparability of groups at baseline, reliability of exposure measurement over time, completeness of the follow-up period, strategy for addressing participant attrition, and the adequacy of the follow-up duration in the context of the analyzed variables.

Each question was scored according to a four-point scale: “Yes”, “No”, “Unclear”, or “Not Applicable”. Based on the percentage of positive answers, the studies were classified as: high quality (≥75% “Yes” answers), moderate quality (50–74%), or low quality (<50%).

#### 2.6.2. Appraisal Tool for Cross-Sectional Studies

As a complementary tool for cross-sectional studies, the Appraisal Tool for Cross-Sectional Studies (AXIS) was applied [10]. AXIS includes 20 items across four domains: (1) introduction and aims (1 question); (2) methods (10 questions regarding design, sample selection, measurement of exposure and outcomes, control of confounding factors, description of non-responders); (3) results (5 questions regarding sample description, statistical analysis, internal consistency, data reporting, and significance); (4) discussion (3 questions regarding interpretation, limitations, and funding sources). Each question was scored dichotomously: “Yes”, “No”, or “Don’t know/comment”. AXIS was used to validate assessments in cases that were ambiguous or borderline in the JBI evaluation.

### 2.7. Effect Measures

Due to the significant methodological heterogeneity of the included studies, such as different testing protocols, measurement scales, and varying methods of reporting results, a formal quantitative meta-analysis was omitted in this systematic review in favor of a qualitative synthesis. For each performance variable, the sets of effect measures described below were extracted:

- Mean difference between groups: the difference between the mean values for FWD and BL, expressed in the units of the original measurement.
- Standardized mean difference: interpretation according to Cohen’s (1988) [44] thresholds: |d| < 0.20—very small; 0.20–0.49—small; 0.50–0.79—moderate; 0.80–1.19—large; 1.20–1.99—very large.
- Hedges’ g: a standardized measure of effect size with a correction for small sample sizes; extracted in studies with N < 30 or when reported by the authors.
- *p*-values: extracted exactly as reported; for *p*-values < 0.001, *p* < 0.01, or *p* < 0.05, the original format was preserved.
- 95% confidence interval: extracted wherever available.
- Percentage difference: where reported.

For the purpose of the qualitative synthesis, a dichotomous direction of the positional effect was determined for each fitness test in every study:

- FWD > BL: test value significantly higher in forwards.
- BL > FWD: test value significantly higher in backs.
- NS: no statistically significant difference (*p* ≥ 0.05).

It should be emphasized that in the case of speed and agility tests, where a lower score indicates a better result, the direction of the effect was determined according to the direction of the better performance level, rather than the direction of the numerical value.

### 2.8. Synthesis Methods

Following the selection and data extraction process, the publications were categorized according to the motor abilities and physical fitness domains evaluated. For each identified motor trait, a table was created describing the tests used and the comparisons between players in different positions. Due to the large number of studies and the varying tests used to assess motor abilities, a narrative interpretation was employed, and the direction of the difference (backs vs. forwards) was indicated. Owing to the extensive volume of data, it was decided to place the comprehensive comparisons in Appendix A to the article.

Conducting a formal meta-analysis was considered a priori, but it was deemed inappropriate for the accumulated evidence base, for reasons evaluated separately for each fitness domain. Methodological diversity within nominally identical domains was non-random: the studies used different protocols, different ergometers and surfaces, and different normalization conventions, meaning that a pooled estimate would not correspond to any interpretable parameter. Additionally, it should be mentioned that the included research designs were exclusively observational and predominantly did not account for the main confounding factor, which is body mass; a meta-analytic mean of unadjusted between-position differences would therefore constitute a quantitative representation of a contrast largely resulting from body size with spurious precision, which is an inferential error this review aims to avoid. Accordingly, the synthesis was conducted in accordance with the SWiM reporting guidelines. Studies were grouped by fitness domain and, within each domain, by test protocol.

## 3. Results

### 3.1. Study Selection

A total of 37 publications were included in the review (Figure 1). It was decided to modify the selection process due to a potentially lower risk of errors when choosing articles eligible for analysis. Each person conducting the selection kept detailed, real-time records explaining why a specific publication could not be qualified, and all criteria as well as the sample counts for individual cases are presented in the figure below. No authors were contacted to provide access to raw data or to the publications. Following the entire process, each author involved in the selection reported significant heterogeneity in the collected data. For this reason, a meta-analysis was omitted. All available data regarding motor skills and physical fitness domains were extracted, excluding data contained within graphics. Finally, a deduplication process was performed by one of the authors after receiving all qualified publications from each of the team members. The entire process is illustrated schematically in Figure 1.

### 3.2. Study Characteristics

Thirty-seven publications published between 1996 and 2025 were included in the narrative synthesis. The total sample size amounted to over 2000 male players aged from 14 years (junior categories) to over 28 years, whereby only adult players (≥18 years) were included in the position-related analyses in accordance with the inclusion criteria. Sample sizes in individual studies ranged from 15 [11] to 510 players [12]. The studies were conducted in 15 countries across four continents: in Australia [13,14,15,16,40], New Zealand [1,12,17,18,19,20,21], England [5,11,22,23,24,25,26,27], Wales [3,28,29], Scotland [45], Spain [30,31], France [32,41], Italy [42], Portugal [33], the USA [34], Brazil [2], Chile [35], Argentina [36], Japan [37,38], and Tunisia [43]. The included studies covered both codes of rugby: rugby union (RU; n = 28) and rugby league (RL; n = 9). The competition level of the participants varied across a wide range: from amateur players [15,20,31,35], through elite juniors of the U20 category [24,43], sub-elite, semi-professional, and academic players [5,14,19,23,28,29,32,34,37,38], up to professional, elite, and international representative players [1,2,3,11,12,13,16,17,18,21,22,25,26,27,33,36,40,41,42,45].

Methodologically, the vast majority of the analyzed papers were observational cross-sectional studies (n = 33). Four studies were longitudinal in nature: a single-season monitoring study, a multi-season observation, a preseason-period protocol, and an inter-match fatigue-monitoring study [40,41,42,43]. The positional classification used in the evaluated papers can be divided into four schemes:

(1) a division into forwards (FWD) vs. backs (BL), which was dominant in 30 studies;

(2) a three-group division used in rugby league encompassing adjustables, hit-up forwards, and outside backs [16];

(3) a five-group division factoring in tight forwards, loose forwards, inside backs, midbacks, and outside backs [21]; and

(4) a detailed division into individual sub-positions [20,30].

The evaluated domains of motor abilities included: muscle strength (n = 20), muscle power (n = 21), sprint speed (n = 19), aerobic capacity (n = 13), agility/change in direction (n = 9), repeated sprint ability (n = 5), and anthropometric characteristics as background data for the entire analysis (n = 27). The most frequently utilized tests were: 1RM squat, 1RM bench press, countermovement jump (CMJ), 10–40 m sprint, Yo-Yo Intermittent Recovery Test, and the agility *t*-test.

### 3.3. Risk of Bias in Studies

The overall methodological quality of the included studies was high or moderately high. All 37 publications scored ≥ 70% positive ratings, which qualified them for inclusion in the analysis. Seventeen publications achieved the maximum score of 100% in the JBI appraisal [1,2,5,12,14,16,17,21,23,25,26,27,33,36,39,42,43], while the remaining studies scored 75% with typical limitations in the domains related to confounding factors (Figure 2).

At the level of individual JBI Cross-Sectional domains, all 33 studies obtained a positive rating in questions Q1–Q4, Q7, and Q8. However, methodological gaps concerned two domains related to confounding factors: in question Q5 (identification of confounding factors), only 48% of the studies obtained a rating of Y, and in question Q6 (strategies for dealing with confounding factors), only 45%, with 33% of the studies receiving a rating of N. This means that more than half of the included papers did not formally control for variables such as age, competition level, training experience, or the phase of the season.

In the JBI Cohort appraisal for the 4 longitudinal studies, two studies achieved a score of 100% [40,42,43] achieved a score of 82% with caveats in the domains regarding the completeness of follow-up (Q9, Q10) [40], and ref. [41] reached a score of 73% with caveats regarding the identification of confounding factors and strategies for dealing with incomplete data [41]. Validation with the AXIS tool for 9 publications with ambiguous JBI appraisals confirmed their inclusion in the review. All of them scored ≥ 76% (range 76–94%), and the most common limitation was the lack of justification for sample size (Q3 AXIS; [11,13,18], which reflects the difficulty in recruiting large samples of elite players typical for sports science research.

The results of the RoB assessment were visualized in the form of traffic-light plots (Figure 2, Figure 3 and Figure 4). The graphics were modified and converted for data presentation purposes in accordance with Cochrane recommendations [46,47] and PRISMA 2020 guidelines [7]. No publication was excluded due to low methodological quality; however, the identified limitations, particularly the incomplete control of confounding factors, were taken into account in the interpretation of the results and in the discussion.

### 3.4. Muscle Strength

Muscle strength was assessed in 20 included studies, most frequently utilizing 1RM bench press and 1RM squat tests, complemented by isometric measurements (IMTP, isometric squat), isokinetic measurements, and derivative tests (chin-up, power clean, leg press, prone row). Comparative analysis revealed a repeatable pattern: forwards achieved higher values of absolute strength, whereas relative values were comparable between positions or higher in backs.

In the scope of 1RM bench press, the vast majority of studies demonstrated an advantage for forwards. In elite and international players, the differences were statistically significant: ref. [33] reported a difference of 109.5 vs. 98.3 kg (*p* = 0.012; ES = 0.55) [33], ref. [1] 146.1 vs. 135.0 kg (*p* = 0.02; ES = 0.81) [1], and ref. [17] 143.6 vs. 130.6 kg (*p* < 0.05). In sub-elite and academic player populations, the differences were more noticeable: La [34]—121.1 vs. 89.5 kg (d = 1.17), ref. [23]—142 vs. 129 kg (d = 0.98), and ref. [19]—133.9 vs. 110.9 kg (ES = 0.96). A similar direction was observed in professional players. Meanwhile, in amateur and junior players, the differences were smaller or non-significant: ref. [31] showed no significant differences (95.0 vs. 100.5 kg; *p* = 0.456). An exception was elite-level juniors, in whom [43] demonstrated a significant advantage for forwards (105.9 vs. 84.0 kg; *p* < 0.05).

An analogous pattern was observed for 1RM squat. At the elite and international level, the differences were as follows: ref. [33]—233.3 vs. 202.4 kg (*p* = 0.015; ES = 1.03), ref. [1]—197.2 vs. 178.1 kg (*p* = 0.02; ES = 0.81), ref. [13]—193.1 vs. 165.0 kg (ES = 2.30), and ref. [17]—197.7 vs. 181.9 kg (*p* < 0.05). At the sub-elite level, La [34] observed a large difference (164.6 vs. 108.5 kg; d = 1.42), and ref. [23]—204 vs. 191 kg (d = 0.74). In juniors, the differences were non-significant, whereas in amateurs, ref. [35] observed an advantage for players in forward positions (153.4 vs. 134.6 kg; *p* = 0.033), and ref. [31] noted an inverse, statistically non-significant trend (195.3 vs. 209.0 kg).

A crucial observation concerns relative strength (1RM/BM). After normalization to body mass, the direction of the differences reversed in favor of players in the backs position. Ref. [17] showed that after allometric scaling, the advantage of forwards in absolute strength measurements disappeared, and in the case of 1RM squat/BM, backs achieved significantly higher values (2.0 vs. 1.8; *p* < 0.01). This observation was confirmed by ref. [23] for 1RM BP/BM (1.35 vs. 1.24; *p* = 0.036; d = 0.70) and 1RM SQ/BM (2.00 vs. 1.79; *p* = 0.001; d = 1.13), ref. [11] for squat/BM (2.18 vs. 1.92; *p* ≤ 0.05), and ref. [2] for 1RM squat/BM (1.84 vs. 1.45; ES = 1.62). An exception is the work of La [34], where an inverse, non-significant direction of differences was observed in academic players (1.8 vs. 1.5; d = 0.85) (Table S1).

### 3.5. Muscle Power

Muscle power was assessed in 21 studies, primarily utilizing the countermovement jump (CMJ) test, as well as the squat jump (SJ), loaded jump squat (JS), bench throw (BT), drop jump (DJ), and standing long jump (SLJ). The analysis revealed a dichotomy in the results depending on whether absolute values, relative values, or jump height were compared.

In terms of CMJ height, an advantage in achieved results was observed for backs over forwards. In elite and international players, the differences were significant: ref. [26] reported a higher jump height in backs in a cohort of 104 RL players (0.36 vs. 0.34 m; *p* = 0.025; g = 0.44), and ref. [27] reported an analogous result in a cohort of 54 players (*p* = 0.03; d = 0.60). Ref. [22] observed a significant advantage for backs in Superleague players (40.3 vs. 37.3 cm; *p* < 0.05), and ref. [38] reported the largest difference to date in academic players in Japan (58.2 vs. 48.1 cm; *p* < 0.01). Ref. [23] confirmed this direction of differences in a sub-elite population (44.82 vs. 40.83 cm; d = 0.75), and ref. [43] noted it in elite-level juniors (32.79 vs. 29.58 cm; *p* < 0.05). Ref. [2] presented a detailed analysis in the form of effect sizes, demonstrating a probable advantage for backs in both the SJ (ES = 1.25) and CMJ (ES = 1.00). In papers conducted at the amateur and junior levels, the differences were generally non-significant. Ref. [30] expanded the positional classification to five sub-groups, demonstrating the lowest values in front row players (34.95 ± 7.97 cm) and the highest in back three players (Table S2).

The opposite direction of differences was observed regarding peak power. Ref. [28] demonstrated higher power in forwards (2444 vs. 2172 W; *p* = 0.012), [22] showed higher power in a loaded 40 kg jump (2106 vs. 1709 W; *p* < 0.05), ref. [11] noted higher power in the SJ (5659 vs. 4740 W; *p* < 0.001), ref. [17] found higher SJ power (5916 vs. 5339 W; *p* < 0.05), ref. [23] reported 5590 vs. 5020 W (d = 1.11), and ref. [1] recorded 5151.8 vs. 4512.1 W (ES = 1.15). Ref. [16] showed that in the three-group classification, outside backs and hit-up forwards achieved the highest peak power (5781 and 5862 W, respectively), while adjustables achieved the lowest (4985 W; *p* < 0.05).

After normalizing power relative to body mass, the pattern of differences reversed. Ref. [17] demonstrated higher relative SJ power in backs (58.9 vs. 53.4 W/kg; *p* < 0.05), ref. [22] confirmed this for loaded JS/BM (20.71 vs. 19.91 W/kg; *p* < 0.05), and ref. [26] reported significantly higher values in backs for both PPmean (31.5 vs. 29.1 W/kg; *p* = 0.004) and PPpeak (54.4 vs. 51.3 W/kg; *p* = 0.002). Ref. [1] observed a similar direction of changes (47.4 vs. 44.7 W/kg; ES = 0.38, NS), ref. [28] found a trend on the verge of significance (26.14 vs. 24.10 W/kg; *p* = 0.051), and ref. [16] noted the highest values in outside backs (62.4 W/kg) compared to hit-up forwards (52.2 W/kg; *p* < 0.05).

### 3.6. Speed

Sprint speed was assessed in 19 studies, utilizing measurements of times over distances ranging from 10 to 50 m as well as calculated maximum sprint speed (MSS). The pattern of positional differences in terms of speed was one of the most noticeable in this review; players in back positions were faster than forwards in almost all included studies, regardless of the competition level and country. In the scope of the 10 m sprint, the majority of studies confirmed an advantage for backs. In elite players, the differences were large and statistically significant: ref. [1] reported 1.66 vs. 1.78 s (*p* < 0.01; ES = 1.99) [1], ref. [23] in a sub-elite population—1.68 vs. 1.78 s (*p* < 0.001; d = 1.72) [18,23] us elite RU players—1.95 vs. 2.04 s (ES = 0.76) [18], ref. [12] in a large cohort of provincial/prof/international (n = 510)—1.69 vs. 1.78 s (*p* < 0.05) [12]. Among juniors and young players, the pattern was as follows: ref. [24] reported 1.99 vs. 2.06 s (*p* = 0.011) [24], ref. [43]—1.69 vs. 1.76 s (*p* < 0.05) [43], ref. [29] in a sub-elite population—1.9 vs. 2.4 s (*p* < 0.05) [29]. Ref. [36] demonstrated the continuity of this pattern across all age categories, where backs were faster than forwards [36]. Exceptions remain the papers by refs. [15,22], where the differences were non-significant, and refs. [15,22,33], in which the lack of significance applied only to absolute values; after normalization to body mass, the differences across all distances were significant with moderate effects (ES = 0.75–0.82). In the scope of the sprint over a distance of 20 m, ref. [1] reported 2.88 vs. 3.11 s (*p* < 0.01; ES = 1.90) [1], ref. [24]—3.26 vs. 3.39 s (*p* = 0.002) [18,24]—3.19 vs. 3.33 s (ES = 0.76) [24], ref. [43]—2.97 vs. 3.15 s (*p* < 0.05) [43], and ref. [36] in seniors—2.98 vs. 3.09 s (*p* < 0.01) [36]. Over longer distances (30–50 m), the differences became more pronounced, suggesting that backs maintain maximum speed better. Ref. [29] observed a difference of almost 1 s over a distance of 40 m (5.8 vs. 6.9 s; *p* < 0.05) [29], ref. [38]—5.41 vs. 5.71 s (*p* < 0.01) [38], ref. [15]—6.45 vs. 6.79 s (*p* < 0.01) [15], ref. [24]—5.55 vs. 5.80 s (*p* = 0.001) [24], and ref. [36] in seniors—5.41 vs. 5.67 s at 40 m and 6.64 vs. 6.99 s at 50 m (both *p* < 0.01) [36]. Ref. [2], using magnitude-based inferences, demonstrated a probable advantage for backs in sprint speed across all analyzed distances (ES = 1.26–1.37) [2]. Ref. [21] expanded the analysis to five sub-positions, demonstrating a decrease in times: tight forwards were the slowest, loose forwards ranked in between, and outside backs proved to be the fastest (ES 0.43–2.48) [21]. In the scope of maximum sprint speed (MSS) and speeds achieved during match conditions, the advantage of backs was analogous. Ref. [38] demonstrated a maximum match speed of 8.06 vs. 7.25 m/s (*p* < 0.01) [38], ref. [35]—6.8 vs. 5.7 m/s (*p* < 0.05) [35], and ref. [36] in seniors recorded 8.42 vs. 7.89 m/s (*p* < 0.01) [36]. Ref. [5], in a kinematic analysis, showed that backs were characterized by shorter foot-ground contact time and higher step frequency, which brought their running profile closer to track-and-field sprinters, whereas forwards generated impulse through longer contact time, which is a less effective strategy for achieving high speed [5]. Players in back positions are significantly faster than forwards across all evaluated distances and all competition levels, which aligns with their playing role that requires explosive running actions. At the same time, forwards generate a higher sprint momentum, which constitutes an indicator of an effective capacity for collisions with an opponent specific to their position (Table S3).

### 3.7. Aerobic Capacity

Aerobic capacity was assessed in 13 studies utilizing indirect and direct measurement methods. The most frequently used tests were the Yo-Yo Intermittent Recovery Test Level 1 (YYIRTL1), Multistage Shuttle Fitness Test (MSFT), measurement of VO_2_peak, and rugby-specific tests: the Bronco test and a 12 min run. In reference to the analysis, players in back positions achieved significantly higher values of aerobic capacity indicators in the majority of studies, and the direction of this difference intensified with an increase in the competition level.

In the scope of measured VO_2_max, an advantage was observed for players in back positions. La [34] reported a VO_2_peak of 54.9 vs. 49.4 mL·kg^−1^·min^−1^ (*p* = 0.017; d = 1.20), ref. [29] based on the MSFT—48.1 vs. 40.3 mL·kg^−1^·min^−1^ (*p* < 0.05), ref. [35]—52.6 vs. 47.5 mL·kg^−1^·min^−1^ (*p* = 0.015; ES = 1.17), where backs obtained values 16.3% higher than forwards, and ref. [14] in sub-elite RL players in a sub-position analysis—46.1 (outside backs) vs. 42.6 (props) ml·kg^−1^·min^−1^ (*p* < 0.05). Ref. [37] utilizing direct spiroergometric measurement confirmed this pattern in academic players in Japan. The only exception remains the work of ref. [15], in which the differences were non-significant in amateur RL players with a low capacity level (38.1 vs. 40.0 mL·kg^−1^·min^−1^).

In the Yo-Yo Intermittent Recovery Test Level 1 (YYIRTL1), all three included studies demonstrated an advantage in parameters for players in the backs position. Ref. [1] reported a large difference of 2237.5 vs. 1516.5 m in elite Super Rugby players (*p* < 0.01; ES = 2.56). Ref. [43] in elite juniors reached 15.43 vs. 14.50 km/h (*p* < 0.05; ES = 1.54), and ref. [3] in international players—1683 vs. 1429 m.

In rugby-specific tests, ref. [41] in professional players demonstrated a significantly shorter Bronco test time in backs compared to forwards (*p* < 0.05). Ref. [38] in a 12 min run observed 2989.8 vs. 2750.3 m in favor of backs (*p* < 0.01), and ref. [20] in a shuttle test in senior-level players demonstrated differences according to sub-positions: inside backs covered 138.2, outside backs 120.4, locks 111.1, and props only 96.3 levels (*p* < 0.001). Ref. [29] expanded the MSFT to different distances (5, 10, 20 m), and in all variants, backs achieved higher results than forwards (*p* < 0.05) (Table S4).

### 3.8. Agility

Agility and change in direction (COD) speed were assessed in 9 included studies, most frequently using the *t*-test, Illinois, Pro-Agility (5-10-5), L-Drill, and Zig-zag tests. The results indicated an advantage for players in back positions in most COD speed tests. It should be noted that after isolating the COD deficit from the overall score, it turned out that significant positional differences result mainly from differences in linear speed, rather than from differences in change in direction technique.

In standard agility tests, ref. [29] demonstrated an advantage for backs in both the *t*-test (10.9 vs. 12.5 s) and the Illinois test (16.8 vs. 17.9 s; both *p* < 0.05). Ref. [43] in elite-level juniors reported 9.90 vs. 11.02 s in the *t*-test (*p* < 0.05; ES = 0.74). Ref. [14] in a sub-elite RL population showed significant differences in agility tests between props and hookers/halves (6.36 vs. 5.93 s; *p* < 0.05), and ref. [20] observed a direction of differences where inside backs were faster than props: 11.5 vs. 12.5 s, which, however, did not reach statistical significance. La [34] in academic players showed no significant differences in the *t*-test and Pro-Agility (*p* > 0.05), despite a moderate effect size (d = 0.62). Ref. [39] in sub-elite senior RL players also found no significant differences in an agility test.

An expanded analysis was presented by ref. [2], utilizing three different COD tests (Pro-Agility, L-Drill, Zig-zag). In terms of COD speed itself, they demonstrated an advantage for backs across all three tests: Pro-Agility ES = 1.67, L-Drill ES = 2.12, and Zig-zag ES = 2.40. Simultaneously, the authors calculated the so-called COD deficit, i.e., the difference between linear sprint time and the COD test time for the same distance. In relation to the COD deficit, no significant positional differences were found (ES = 0.47–0.55) (Table S5).

### 3.9. Anaerobic Capacity and Repeated Sprint Ability (RSA)

Anaerobic capacity and repeated sprint ability (RSA) were assessed in five included studies, utilizing repeated sprint protocols that differed in the number of repetitions, distance, and recovery duration. The gathered data indicate that backs achieved better results in terms of total sprint time and distance covered in the RSA protocol, whereas indicators representing fatigue did not differ significantly between positions. Ref. [38] demonstrated that backs covered a significantly greater distance in the RSA test than forwards (640.4 vs. 607.4 m; *p* < 0.01), while the change in lactate concentration (Δ La) after the test did not differ between positions (16.1 vs. 13.8 mmol/L; *p* > 0.05). Ref. [12], conducting an analysis on a large sample (n = 510) using the Rugby-Specific Repeated-Speed test, reported a lower mean sprint time in backs compared to forwards, yet the percentage fatigue index did not differ between positions (2.8 vs. 2.6%; *p* > 0.05). Ref. [20] showed that the fatigue index in a 30 m repeated sprint test was lower in inside backs compared to locks (39.8 vs. 54.3; *p* = 0.039), suggesting a better tolerance for repeated efforts in backs players (Table S6).

### 3.10. Anthropometric Characteristics Comparison

Anthropometric characteristics were reported in 27 out of 37 included studies. The most frequently analyzed parameters were body mass (BM), body height, body fat percentage (BF%), fat-free mass (FFM), and the sum of skinfold thicknesses. Anthropometric data serve as an interpretative background in this review because, as demonstrated in previous sections, body mass is a key explanatory factor for positional differences in absolute strength, absolute power and sprint momentum.

Body mass was the most consistent determinant of positional differences, with every single one of the 24 studies providing descriptive data divided by position showing an advantage for forwards over backs, without exception and regardless of the competition level. The differences ranged from approximately 10 kg in elite juniors (ref. [24]: 90.1 vs. 82.8 kg, NS) and amateurs (ref. [15]: 90.8 vs. 79.7 kg; *p* < 0.01), through 15–20 kg in sub-elite and academic players (La [34]: 90.5 vs. 73.7 kg; d = 1.60; ref. [38]: 96.1 vs. 80.3 kg; *p* < 0.01), up to 20–25 kg in elite and international players (ref. [1]: 116.5 vs. 95.9 kg; ES = 2.10; ref. [2]: 108.3 vs. 84.0 kg; ES = 2.27; ref. [3]: 115.8 vs. 94.5 kg). Ref. [5] presented these data in a broader context within a kinematic analysis, comparing rugby players with track-and-field sprinters: forwards weighed 111.6 kg, backs 88.6 kg, and sprinters only 75.7 kg.

In terms of body fat content, forwards exhibited a significantly higher BF% than backs in academic players (ref. [38]: 19.8% vs. 15.5%; *p* < 0.01; ref. [34]: 12.6% vs. 8.8%; d = 1.10), elite players (ref. [12]: 14.0% vs. 10.5%), semi-professional players (ref. [32]: 18.1% vs. 14.8%), and amateurs (ref. [35]: 25.1% vs. 22.5%; *p* = 0.018). The only exception was the study by ref. [15] in amateur RL players (19.9% vs. 17.5%; NS). Fat-free mass was also higher in forwards (ref. [12]: 94.1 vs. 83.1 kg; *p* < 0.05), which indicates that the body mass advantage of forwards results from both higher muscle mass and higher fat mass, aligning with the position-specific match demands of their game (Table S7).

### 3.11. Directional Synthesis of Differences Relative to Playing Position

The pattern of positional differences in rugby is consistent and highly repeatable in the literature, regardless of the game code (RU vs. RL), country (≥15 countries), competition level, and age. Players in forward positions are characterized by a greater body mass, higher BF%, higher absolute strength, and higher absolute power, which is consistent with the physiological demands of their role on the pitch. Players in back positions are faster, exhibit higher relative CMJ power, higher jumping ability, better aerobic capacity, and better agility, which aligns with their on-field role. After normalization to body mass through allometric scaling, positional differences are strongly attenuated or reversed, which may indicate that the existing positional differences are largely an effect of the difference in BM. The COD deficit does not differ relative to position; the advantage of BL in agility stems from their superior linear speed rather than differences in change in direction technique [2]. Positional differences are least pronounced in younger players and amateurs, and strongest in elite/international players, indicating that differences increase with training specialization and selection at higher levels of competition (Table 2).

## 4. Discussion

Throughout this Discussion, a distinction is maintained between evidence-based statements and interpretive explanations. Statements describing the direction and magnitude of positional differences are taken directly from the extracted data of the included studies. In contrast, the physiological and biomechanical mechanisms proposed to account for these differences are interpretive and were not directly tested within the included studies.

### 4.1. Muscle Strength: Absolute and Body-Mass-Normalized Differences

This systematic review demonstrated a pattern where forwards achieved higher scores in terms of absolute muscle strength, which was noted in 20 studies published between 1996 and 2025, utilizing 1RM bench press, 1RM squat, isokinetic dynamometry, and a range of other derivative tests. This result aligns with the requirements of position-specific tasks for forwards, particularly playing in scrums and contact-oriented play with the ball, where the ability to generate high absolute force directly conditions the effectiveness of gaining “meters” and breaking the defensive line.

In the 1RM bench press, the advantage of forwards was statistically significant in the majority of studies at the elite and sub-elite levels: ref. [33] reported 109.5 vs. 98.3 kg (*p* = 0.012; ES = 0.55), ref. [1]—146.1 vs. 135.0 kg (*p* = 0.02; ES = 0.81), ref. [17]—143.6 vs. 130.6 kg (*p* < 0.05), La [34]—121.1 vs. 89.5 kg (d = 1.17), ref. [23]—142 vs. 129 kg (d = 0.98), and ref. [19]—133.9 vs. 110.9 kg (ES = 0.96). At the professional level, ref. [13] recorded one of the largest effect sizes in the entire data set for the 1RM squat: 193.1 vs. 165.0 kg (ES = 2.30), which suggests that at the highest level of competition, specialization in terms of tasks for the assigned player position regarding lower limb strength expressed in absolute values is particularly pronounced. The transition from small or non-significant differences at the amateur level to large effects in elite and professional groups suggests the existence of a correlation with the athletic level, reflecting the cumulative impact of position-specific targeted training and long-term processes of athletic selection.

However, the most important conclusion related to the achieved muscle strength values is the reversal of the direction of positional differences after normalizing the results relative to body mass. When 1RM values are expressed relatively (1RM/BM), backs achieve results that are at least comparable, and often better, than forwards: ref. [17] demonstrated a significantly higher relative strength in the squat in backs (2.0 vs. 1.8 1RM/BM; *p* < 0.01) and showed that allometric scaling completely eliminates the advantage of forwards in absolute values; ref. [23] reported 1RM BP/BM of 1.35 vs. 1.24 (*p* = 0.036; d = 0.70) and 1RM SQ/BM of 2.00 vs. 1.79 (*p* = 0.001; d = 1.13) in favor of backs; ref. [11] noted a squat/BM of 2.18 vs. 1.92 (*p* ≤ 0.05) in backs; ref. [2]—1RM squat/BM of 1.84 vs. 1.45 (ES = 1.62). The convergence of these observations leads to the conclusion that the described advantage of forwards in terms of absolute strength is largely a function of greater body mass, rather than a greater capacity to generate force in a short time.

In the context of practical recommendations, strength and conditioning coaches should use relative strength indicators or employ allometry when comparing physical development between positional groups, as operating with absolute values overestimates the evaluation of strength preparation of forwards in relation to backs. The lack of normalization relative to body mass increases the risk of misclassifying overly heavy forwards as physically more efficient in certain parameters, while simultaneously underestimating the level of strength preparation of backs.

Additionally, attention should be paid to isometric tests. Ref. [22] reported higher peak force in the isometric squat in forwards, but after calculating per body mass, the differences were attenuated. The data in the scope of isokinetic measurements by ref. [3] additionally showed that although the peak torque of knee extensors was higher in absolute values in forwards, it did not constitute a predictor of key match performance indicators after accounting for position, which suggests that positional demands are better reflected by tests of sport-specific movements than by isolated torque measurements in a given joint.

### 4.2. Muscle Power: Absolute Output and Relative Performance

Data regarding muscle power demonstrated a duality of results very close to the pattern observed in the domain of muscle strength, which can potentially be explained by the same factor: body mass. In terms of absolute values, forwards achieved higher peak power in loaded jump tests, squat jumps, bench throws, and CMJ: ref. [28]—2444 vs. 2172 W (*p* = 0.012); ref. [22]—2106 vs. 1709 W (*p* < 0.05) in a loaded 40 kg jump; ref. [11]—5659 vs. 4740 W in the SJ (*p* < 0.001); ref. [17]—5916 vs. 5339 W (*p* < 0.05); ref. [23]—5590 vs. 5020 W (d = 1.11); ref. [1]—5151.8 vs. 4512.1 W (ES = 1.15). After normalization relative to body mass, the direction of the differences reversed in favor of backs. Ref. [17] demonstrated higher relative SJ power in backs (58.9 vs. 53.4 W/kg; *p* < 0.05); ref. [22] confirmed this for JS/BM (20.71 vs. 19.91 W/kg; *p* < 0.05); ref. [26] JSCR) [26] recorded higher values of PPmean (31.5 vs. 29.1 W/kg; *p* = 0.004) and PPpeak (54.4 vs. 51.3 W/kg; *p* = 0.002) in backs within a cohort of 104 rugby league players; ref. [16] showed the highest relative peak power in outside backs (62.4 W/kg) compared to hit-up forwards (52.2 W/kg; *p* < 0.05).

Data regarding countermovement jump (CMJ) height, which in practice serves as a measure of relative lower limb power, provide evidence demonstrating an advantage in terms of developed lower limb power in backs. In 9 out of 15 studies, backs jumped higher, including ref. [26,48] (d = 0.60; *p* = 0.03), ref. [27,48] (g = 0.44; *p* = 0.025), ref. [22,48] (40.3 vs. 37.3 cm; *p* < 0.05), ref. [38,48] (58.2 vs. 48.1 cm; *p* < 0.01), ref. [23,48] (44.82 vs. 40.83 cm; d = 0.75), ref. [2,48] (SJ ES = 1.25, CMJ ES = 1.00), and ref. [43,48] (32.79 vs. 29.58 cm; *p* < 0.05 in U20). An analysis by ref. [30,48] additionally showed that the lowest values are obtained by front-row forwards (34.95 ± 7.97 cm), and jump height increases toward the back-row formation. This advantage is most parsimoniously explained by the lower body mass of backs, which reduces the mechanical cost of vertical displacement, together with a plausible contribution of stretch-shortening-cycle efficiency; a role of muscle fiber-type distribution cannot be excluded but was not directly assessed in any of the included studies [13,31,48].

Studies in which no significant differences were found in CMJ [11,17,18,22,28] mainly involved amateur, junior, or sub-elite populations, which is consistent with the observation that positional differentiation in explosive power intensifies with an increase in competitive level and specialization. An exception is La Monica [34], where a statistically non-significant trend in favor of forwards was observed in relative strength within a US collegiate population (1.8 vs. 1.5 1RM squat/BM; d = 0.85), which might be associated with a non-representative structure of the US collegiate population.

### 4.3. Sprint Speed

Sprint speed demonstrated the most statistically significant positional differentiation among all domains of physical fitness. In the majority of studies, backs achieved better times across all analyzed distances (10–50 m) and at all competition levels, across 15 countries and over a span of nearly three decades. The largest effect sizes concerned the 10 m sprint: ref. [1]—1.66 vs. 1.78 s (*p* < 0.01; ES = 1.99), ref. [23]—1.68 vs. 1.78 s (*p* < 0.001; d = 1.72). Similar differences were observed over a distance of 20 m (ref. [1]: 2.88 vs. 3.11 s; ES = 1.90 [1,24]: 3.26 vs. 3.39 s; *p* = 0.002; ref. [43]: 2.97 vs. 3.15 s [43]), and with increasing distance, the differences widened further.

Over the 30–50 m sections, where players approach their maximum speed, the advantage of backs in maintaining velocity became particularly pronounced. Ref. [29] recorded a difference close to one second over 40 m (5.8 vs. 6.9 s; *p* < 0.05); ref. [38]—5.41 vs. 5.71 s (*p* < 0.01); ref. [15]—6.45 vs. 6.79 s (*p* < 0.01) [15,36]—5.41 vs. 5.67 s at 40 m and 6.64 vs. 6.99 s at 50 m (both *p* < 0.01) [36]. These data suggest that the speed advantage of backs is greatest during the maximum speed maintenance phase, which is potentially associated with lower body mass and superior running mechanics.

A sub-positional analysis by ref. [21] in a five-group classification demonstrated the following differences in terms of achieved speed: tight forwards were the slowest, loose forwards were intermediate, and outside backs were the fastest across all distances (d = 0.43–2.48 between the extreme sub-positions) [21]. Ref. [20] reported a similar change relative to playing position for sprint speed, which suggests that this is an inherent trait relative to position in the game rather than an effect of training modifications [20].

### 4.4. Aerobic Capacity and Positional Running Demands

Players occupying backline positions demonstrated higher aerobic capacity across all measurement methods used: direct assessments (VO_2_max/VO_2_peak), Yo-Yo IRTL1, rugby-specific tests (Bronco, 12 min run, MSFT), and submaximal protocols. These differences were most pronounced among elite and professional players. The largest effect size (ES) was reported by ref. [1], where Yo-Yo IRTL1 performance reached 2237.5 vs. 1516.5 m (*p* < 0.01; ES = 2.56) in Super Rugby players, representing an average difference of 32% in distance covered. For VO_2_peak/VO_2_max, backs achieved superior aerobic fitness parameters in five of the six studies: La [34]—54.9 vs. 49.4 mL·kg^−1^·min^−1^ (*p* = 0.017; d = 1.20); ref. [29]—48.1 vs. 40.3 mL·kg^−1^·min^−1^ (*p* < 0.05); ref. [35]—52.6 vs. 47.5 mL·kg^−1^·min^−1^ (*p* = 0.015; ES = 1.17); and ref. [14]—46.1 vs. 42.6 mL·kg^−1^·min^−1^ in a positional analysis of rugby league players. Ref. [37] confirmed this pattern in a Japanese collegiate population, additionally reporting larger cardiac dimensions among backs, consistent with the aerobic demands characteristic of their playing positions.

The lower aerobic capacity observed in forwards may be multifactorial. Specifically, their greater body mass and potentially higher body fat levels increase the energetic cost of exercise, thereby reducing VO_2_max when expressed relative to body mass. Furthermore, players occupying these positions more frequently follow training programs focused on the development of strength and power, which undoubtedly provide a smaller stimulus to the cardiorespiratory system than the traditional running- or interval-based training typically performed by backs. When considering the match activity profile of forwards, they are characterized by frequent short-duration explosive actions and involvement in set-piece situations, which may place less demand on mechanisms associated with cardiorespiratory endurance. Longitudinal data from ref. [43] demonstrated that these differences are already evident at the elite junior level (U20; YYIRTL1 15.43 vs. 14.50 km/h; *p* < 0.05; ES = 1.54), suggesting that the divergence in aerobic fitness begins during the early stages of specialization. Ref. [41] showed that this gap persists despite a standard pre-season preparation period among professional players, indicating that typical pre-season training programs do not fully eliminate position-specific differences in aerobic fitness.

### 4.5. “Change of Direction” Ability and Agility

Agility and change-of-direction (COD) ability were assessed in nine studies using standardized tests, including the *t*-test, Illinois Agility Test, Pro-Agility Test, L-Drill, and Zig-zag Test. Overall, backs achieved faster times in COD speed assessments. Ref. [29] reported significant differences in both the *t*-test (10.9 vs. 12.5 s; *p* < 0.05) and the Illinois Agility Test (16.8 vs. 17.9 s; *p* < 0.05). Similarly, ref. [43] found *t*-test performances of 9.90 vs. 11.02 s among U20 players (*p* < 0.05; ES = 0.74), while ref. [2] demonstrated a clear advantage for backs across all three COD assessments, with large effect sizes ranging from 1.67 to 2.40. However, several studies conducted in sub-elite and amateur populations did not identify significant positional differences ([20,34,39]).

The findings reported by ref. [2] provide an important alternative interpretation of these results. The authors calculated the so-called COD deficit, defined as the difference between linear sprint time and COD test time over an equivalent total distance. This metric is considered an indicator of braking, directional change, and re-acceleration ability independent of linear sprint performance. For COD deficit, no significant positional differences were observed (ES = 0.47–0.55), despite the substantial differences found in COD test times. These findings suggest that the superior performance of backs in agility assessments may be entirely attributable to their greater linear sprint speed rather than to a superior ability to execute directional changes. From a practical perspective, agility tests should not be regarded as standalone measures of movement proficiency when comparing players from different positions. Future research and coaching practice in rugby should therefore incorporate COD deficit calculations alongside conventional COD test times to provide a more accurate assessment of change-of-direction performance.

### 4.6. Repeated Sprint Ability and Anaerobic Capacity

Data on Repeated Sprint Ability (RSA) and anaerobic capacity are the least represented within the literature. Only five studies reported RSA outcomes according to playing position, despite the importance of repeated high-intensity efforts for both forwards and backs. The available evidence suggests that backs generally achieve superior performance in RSA protocols, while no substantial positional differences are observed in fatigue-related indices or metabolic responses.

Ref. [38] reported a greater total distance covered by backs during a standardized RSA test (640.4 vs. 607.4 m; *p* < 0.01), with no significant differences in blood lactate accumulation (ref. [34]Δ: 16.1 vs. 13.8 mmol·L^−1^; *p* > 0.05). Similarly, ref. [12], in the largest sample included (n = 510), demonstrated faster mean sprint times among backs in a rugby-specific repeated sprint test, while the percentage fatigue index remained comparable between positional groups (2.8 vs. 2.6%). Ref. [20] observed a lower fatigue index in inside backs compared with locks (39.8 vs. 54.3; *p* = 0.039) during a repeated 30 m sprint protocol. This was the only study to identify a significant positional difference in fatigue-related outcomes, although this finding may have been partially influenced by the lower body mass of inside backs.

The absence of differences in fatigue indices and lactate responses despite clear differences in sprint times and total distance covered suggests that both positional groups possess a broadly similar capacity to tolerate and recover from repeated maximal efforts. Consequently, performance differences appear to be largely attributable to variations in sprint speed and body mass rather than distinct fatigue resistance mechanisms. However, due to the very limited number of available studies and the absence of position-specific data from laboratory-based anaerobic assessments such as the Wingate test, firm conclusions cannot be drawn. Therefore, Repeated Sprint Ability and anaerobic capacity should be considered a significant gap in the current rugby performance literature.

### 4.7. Anthropometric Context as a Scaling Variable

Anthropometric data from 27 studies form the primary context for all physical fitness comparisons. Body mass proved to be the most variable parameter: across all 24 studies reporting BM by position, forwards were heavier, regardless of level and country. The magnitude of differences increased with performance level, from approximately 10 kg in junior elite and amateur players [15,24], through 15–20 kg at sub-elite and collegiate levels [34,38], up to 20–25 kg at elite and national representative levels [1,2,3]. Body fat percentage was higher in forwards, with differences being significant (ref. [38]: 19.8% vs. 15.5%; [34]: 12.6% vs. 8.8%; ref. [12]: 14.0% vs. 10.5%; ref. [35]: 25.1% vs. 22.5%). Lean body mass was also greater in forwards (ref. [12]: 94.1 vs. 83.1 kg), confirming that the BM advantage encompasses both muscle and fat tissue. These differences are simultaneously a cause and a consequence of position-specific demands and constitute the primary mechanism explaining the superiority of forwards in absolute values of strength, power, and momentum.

### 4.8. Limitations of the Study

When interpreting the results of this review, several limitations must be taken into account. The considerable heterogeneity of testing protocols, including varied sprint distances, jump test variants, fitness assessment methods, and strength testing standards, precluded the application of formal quantitative meta-analysis and necessitated a narrative synthesis of results. Consequently, unified statistical estimates of effect size cannot be provided. More than half of the cross-sectional studies did not identify or control for confounding variables such as age, training experience, or nutritional status, which may have introduced bias into positional comparisons, particularly in samples where forwards and backs also differed in training structure and volume. The binary division into “forwards and backs” used in 30 of the 37 studies is a methodological simplification that potentially masks within-group variability. The results of [21] using a five-group classification demonstrate that, for example, the Number 8 (loose forward) is physically closer in profile to inside backs than to front-row forwards (props), and averaging within a single “FWD” category obscures these differences. The broad time span (1996–2025), being a characteristic feature of high-quality systematic reviews, encompasses a period of substantial transformation in rugby, such as shifts in training trends or the evolution of match loads. It is possible that positional differences have intensified over time; however, due to the descriptive nature of the synthesis, formal testing of secular trends was not feasible.

A further limitation arises from the eligibility restrictions applied during searching. Because eligibility was confined to peer-reviewed original articles published in English, potentially relevant evidence published in other languages or in the gray literature may not have been captured, which may introduce a degree of language and publication bias whose direction cannot be determined from the included data. In addition, outcomes reported only in figures were not extracted and study authors were not contacted for unpublished data, which may have led to the omission of a small number of individual results.

### 4.9. Practical Implications

From a practical standpoint, strength and conditioning coaches should compare physical development across positional groups using performance measures normalized relative to body mass through allometric scaling, if an appropriate exponent is available, rather than using absolute values alone. Training programs should reflect differences in match demands: for forwards, an emphasis on generating high absolute force and contact capacity; for backs, the development of linear speed, aerobic fitness, and repeated sprint ability. Speed training in rugby should focus primarily on the horizontal component of ground reaction force and running momentum, rather than solely on maximum velocity, which is largely morphologically determined. Backs require periodisation focused on the development of linear speed, high-intensity aerobic fitness, including high volumes of intermittent work such as Yo-Yo testing protocols, and rugby-specific RSA protocols. In the area of talent identification and selection, the findings suggest that positionally differentiated physical profiles should be applied at sub-elite and junior elite levels, as differences in strength, power, sprint, and fitness parameters are detectable as early as the U20 age category and in collegiate populations. Practitioners should routinely employ body-mass–normalized measures, avoiding sole reliance on absolute values, which can potentially favor forwards. Jump height and relative peak power (W/kg) constitute useful neuromuscular readiness indicators that are largely independent of body mass.

### 4.10. Future Recommendations

The findings of this review allow for the identification of several research directions. Studies employing more detailed sub-positional classifications, extending beyond the classical forwards-backs (FWD–BL) division, are clearly needed to enable the development of reference norms for each individual position. Longitudinal studies tracking the course of physical development from junior to professional level are also required, in order to determine what proportion of positional differences results from training and what proportion from selection.

Future studies should report both absolute and normalized values, which will facilitate cross-study comparisons and the construction of positional benchmarks. The COD deficit methodology proposed by ref. [2] should become a standard component of agility assessment, enabling the separation of the influence of linear speed from actual change-of-direction ability.

## 5. Conclusions

To our knowledge, this systematic review provides one of the first comprehensive syntheses of positional differences in physical fitness between forwards and backs in adult male rugby, encompassing all major fitness domains. The following conclusions directly address the review objectives. Forwards demonstrate higher absolute muscular strength and power regardless of competitive level, rugby code, and testing methods applied. However, following normalization relative to body mass, this advantage is systematically attenuated or reversed in favor of backs, indicating that body mass is the principal scaling factor in comparisons of absolute strength and power between positional groups. With respect to backs, they demonstrate higher sprint speed across all distances, greater aerobic fitness, greater CMJ height, and higher relative muscular power (W/kg). These advantages are observed across various competitive levels and in both rugby codes, with their magnitude increasing progressively from amateur to elite and professional levels. The methodological quality of the 37 included studies was predominantly high or moderately high; however, insufficient control of confounding variables and the widespread use of the forwards–backs division limit the capacity for developing positional fitness benchmarks.

## Figures and Tables

**Table 1:** Inclusion and exclusion criteria according to PICOs.

PICOs	Inclusion Criteria	Exclusion Criteria
P—Population	- Male rugby players aged ≥ 18 years - Rugby union (15-a-side) and/or rugby league (13-a-side) - Any competitive level: amateur, sub-elite, semi-professional, professional, elite, or international - Any country or region	- Female players; - Players aged < 18 years (unless mean age of sample is ≥18 with SD not crossing below 17); - Rugby sevens exclusively (distinct game model); - Mixed-sex samples where male data cannot be extracted separately; - Touch rugby, tag rugby, or wheelchair rugby
I—Intervention/Exposure	- Playing position as the independent variable - At minimum, a binary comparison (forwards vs. backs) must be reported - More granular positional groupings are acceptable	-
C—Comparison	- Between at least two positional groups within the same study sample - Groups must be defined by playing position (not by playing level, selection status, or other criteria)	-
O—Outcomes	(At least one must be reported with descriptive statistics by positional group): - Muscular strength - Muscular power - Sprint speed - Aerobic/endurance capacity: VO_2_max or VO_2_peak - Change in direction/agility - Repeated sprint ability - Anaerobic capacity	- Studies reporting only anthropometric or body composition variables without any physical fitness test; - Studies reporting only GPS/accelerometer match-demand data without laboratory or field-based fitness testing; - Studies reporting only game statistics or technical skill assessments
S—Study type	- Observational: cross-sectional, prospective or retrospective cohort, comparative designs - Intervention studies: included only if baseline (pre-intervention) data comparing positions is reported and extractable - Longitudinal studies: included if positional comparison data at any time point is extractable	Systematic reviews and meta-analyses; case reports, letters to the editor, conference abstracts, book chapters; intervention studies without baseline values comparing positions;

**Table 2:** Directional synthesis divided by basic motor tests and characteristic motor traits.

	Number of Studies	Dominant Direction	Supporting Studies	Opposing Studies/NS	Conclusion
Absolute strength (1RM BP, SQ, chin-up, leg press, IMTP)	14	**FWD > BL**	[13,17,23,43] (BP); [1,12,19,33,34,35,38] (SQ); [22] (isom.); [16]	[31] (NS); [24] (NS); [43] (SQ NS); [38] (clean NS); [35] (BP NS)	FWD stronger at higher levels of competition; in juniors and amateurs, the differences were smaller or NS. The largest ES (d > 1.0) in studies on elite players.
Relative strength (1RM/BM)	8	**BL > FWD or NS**	[11] (SQ); [17] (BP, SQ); [2] (SQ); [23] (BP, SQ)	[24] (NS); [34]; [19] (NS); [35] (NS)	After normalization to BM, most differences reverse in favor of BL. Allometric scaling eliminates positional differences. This suggests that the observed differences in strength are mainly an effect of body mass.
Absolute CMJ/SJ power (W)	8	**FWD > BL**	[22,28]; [11]; [1,17,23]; [16]	—	FWD generate higher absolute power in loaded tests, secondary to a higher BM.
Relative CMJ/SJ power (W/kg)	8	**BL > FWD**	[28]; [22]; [17]; [26]; [1]; [16]	—	After BM normalization, the pattern of differences reverses; BL generate higher relative power, which translates into better vertical jumps.
Vertical jump height (cm)	13	**BL > FWD**	[2,22,23,26,30,41,43]; [26]; [38]	[15] (NS); [39]; [24] (NS); [34] (NS); [20] (NS); [35] (NS)	BL higher jumps in 9/15 studies, significant mainly in the elite cohorts. In juniors and amateurs, the differences were smaller or NS.
SPRINT 10 m (s)	14	**BL > FWD**	[1,12,14,18,23,24,29,36,42,43]	[22] (NS); [15] (*p* = 0.07); [33] (NS abs.)	BL faster over 10 m in 11/14 studies. The largest ES in the elite cohorts (Posthumus ES = 1.99; Hart d = 1.72).
SPRINT 20 m (s)	8	**BL > FWD**	[1,14,18,24,36,43]	[22] (NS); [33] (NS abs.)	BL are consistently faster; in RL, the differences tend to be smaller.
SPRINT 30–50 m (s)	11	**BL > FWD**	[2,14,15,20,21,24,29,36,38]	[34] (NS); [35] (NS); [33] (NS abs.)	Differences increase with distance. BL maintain Vmax better.
MAX SPRINT SPEED (MSS, m/s)	6	**BL > FWD**	[38]; [5,36]; [35]	[18]	BL achieve a higher MSS, close to track-and-field sprinters.
Sprint momentum (kg·m/s)	3	**FWD > BL**	[2]; [36,42]	—	FWD possessed a higher body momentum (BM × v), which is a key KPI for players in contact positions.
VO_2_max/VO_2_peak	6	**BL > FWD**	[14,29,37]; [34,35]	[15] (NS)	BL higher VO_2_max/peak in most studies; the difference increases with the competition level.
Yo-Yo IRTL1 (m or km/h)	3	**BL > FWD**	[1,3,43]	—	All 3 studies: BL higher Yo-Yo. The largest ES in elite players ([1]; ES = 2.56).
Agility/COD velocity (*t*-test, Illinois, Pro-agility, L-Drill, Zig-zag)	8	**BL > FWD**	[2,14,29,43]	[14]; [34]; [20]	Backs are faster in COD speed tests; however, the COD deficit does not differ positionally. The effect stems from linear speed, not from “change of direction” technique.
COD deficit	1	**NS**	—	[2] (NS)	Only study; the COD deficit does not differ between positions, which indicates that the positional effect in agility stems from linear speed.
RSA—total time/distance	4	**BL > FWD**	[12,20,38]	—	BL better in RSA, but the fatigue index (%) and metabolic response were sometimes similar between the given positions.
Body mass (BM, kg)	24	**FWD > BL**	All 24 studies with BM data by position	—	No study demonstrated BL ≥ FWD in BM. FWD were on average 10–25 kg heavier. In the elite cohorts, the differences were the largest (Posthumus ES = 2.10; Freitas ES = 2.27).
BODY FAT (%)	6	**FWD > BL**	[32]; [12,34,35,38]	[15] (NS)	FWD possessed a higher BF%. A similar pattern was observed across all competitive levels.

FWD–forwards—forwards (props, hookers, locks, flankers, no. 8); BL–backs—backs/backline (halfbacks, fly-halves, centers, wings, fullbacks); RL—Rugby League (13-a-side); NS—statistically non-significant (*p* > 0.05); COD—change in direction; ES—effect size; KPI—key performance indicators; RSA—repeated sprint ability; colors: pink—FWD > BL (forwards higher score); blue—BL > FWD (backs higher score); gray—NS; no significant positional differences.

## Data Availability

The original contributions presented in the study are included in the article; further inquiries can be directed to the corresponding authors.

## Supplementary Materials

The following supporting information can be downloaded at: https://www.mdpi.com/article/10.3390/jfmk11030313/s1, Table S1: Muscle strength: comparison of forwards and backs in the included studies; Table S2: Muscle power (CMJ, SJ, JS, BT): comparison of forwards and backs in the included studies; Table S3: Sprinting speed: comparison of forwards and backs in the included studies; Table S4: Aerobic capacity: comparison of forwards and backs in the included studies; Table S5: Agility and change of direction (COD): comparison of forwards and backs in the included studies; Table S6: Repeated sprint ability and anaerobic capacity: comparison of forwards and backs in the included studies; Table S7: Anthropometric characteristics: comparison of forwards and backs in the included studies.

## Abbreviations

Playing positions	
FWD/F	forwards-attacking forwards (props, hookers, locks, flankers, no. 8)
BL/B	backs-defensive line (halfbacks, fly-halves, centres, wings, fullbacks)
HUF	hit-up forwards-collision forwards (Rugby League)
ADJ	adjustables/halves-playmaking positions (Rugby League)
OB	outside backs-wide defenders (wings, fullback) (Rugby League)
FR	front row-first line (props, hooker) (Rugby Union)
SR	second row-second line (locks) (Rugby Union)
BR	back row-third line (flankers, no. 8) (Rugby Union)
Rugby type/competition level	
RU	Rugby Union (15-a-side)
RL	Rugby League (13-a-side)
NRL	National Rugby League (Australia, RL)
Super Rugby	elite international RU league (Southern Hemisphere)
Superleague	elite RL league (England)
CoE	Centre of Excellence (talent development academy)
GF/NSGF	Grand Final players/Non-Selected for Grand Final (NRL)
Strength tests	
1RM	one repetition maximum
BP	bench press
SQ	squat
PC	power clean
IMTP	isometric mid-thigh pull
PF	peak force
PRFD	peak rate of force development
FR (1RM/BW)	force ratio = 1RM/body weight (relative strength)
Isom. SQ	isometric squat
Power/jump tests	
CMJ	countermovement jump
SJ	squat jump (no countermovement)
JS	jump squat (loaded)
DJ/DJ45	drop jump/drop jump from 45 cm
BT	bench throw (ballistic upper-body)
VJ	vertical jump
SLJ	standing long jump
HTJ	horizontal triple jump
PP/PPpeak	peak power
PPmean	mean propulsion power
RSI/RSImod	reactive strength index/modified
JH	jump height
CMB/COB	countermovement bench throw/concentric-only bench throw
Fmax/Vmax	maximum force/velocity (from F-V profile)
Speed/cod tests	
MSS/vmax	maximum sprint speed (m/s)
F0	theoretical maximum force
Fopt	optimal force for peak power
Pmax	maximal horizontal power (W/kg)
F-V profile	force-velocity profile (sprint)
COD	change of direction
COD deficit	COD deficit = difference between linear sprint and COD over the same distance
T-test	T-test (T-shaped change of direction, 36.5 m)
Pro-agility (5-10-5)	Pro-Agility test (5–10–5 m)
L-Drill	L-Drill change-of-direction test
Illinois	Illinois agility test
Zig-zag	zig-zag test (maneuverability)
CM velocity	centre of mass velocity
Aerobic fitness	
VO_2_max/VO_2_peak	maximal/peak oxygen uptake (mL·kg^−1^·min^−1^)
Yo-Yo IRTL1/IRTL2	Yo-Yo Intermittent Recovery Test Level 1/2
MSFT/MST	Multistage Shuttle Fitness Test/Multistage Test
VIFT/30-15 IFT	final speed in 30-15 Intermittent Fitness Test
CPET	cardiopulmonary exercise testing
Bronco test	1200 m shuttle run test (RU fitness)
12-min run	Cooper test-distance covered in 12 min
HSR	high-speed running
Rsa/anaerobic capacity	
RSA	repeated sprint ability
RSAtot	total time in RSA test
RSAdec	RSA decrement (% speed decline between sprints)
La/Δ La	blood lactate/change in lactate (mmol/L)
Anthropometry	
BM	body mass (kg)
BF%/%Fat	body fat percentage
FFM/LBM	fat-free mass/lean body mass
Σ4-SF/Σ6-SF/Σ8-SF	sum of 4/6/8 skinfolds (mm)
CSA	cross-sectional area (muscle)
MT	muscle thickness (ultrasound)
PA	pennation angle (muscle fibre)
DXA	dual-energy X-ray absorptiometry
Statistics	
M ± SD	mean ± standard deviation
95% CI	confidence interval (95%)
ES	effect size
d (Cohen)	Cohen’s d (effect size); 0.2 = small, 0.5 = medium, 0.8 = large
g (Hedges)	Hedges’ g (effect size with small-N correction)
NS	not statistically significant (*p* > 0.05)
*p*	probability of Type I error
*	*p* < 0.05
**	*p* < 0.01
***	very likely (MBI)/*p* < 0.001
****	almost certainly (MBI)
MBI	magnitude-based inferences
ICC	intraclass correlation coefficient (reliability)
KPI	key performance indicators (match performance)
Testing periods (longitudinal studies)	
T0/T1/T2	baseline/mid-season/end-of-season time point
CK/LDH	creatine kinase/lactate dehydrogenase (muscle damage markers)
BDNF	brain-derived neurotrophic factor
Table markers	
“-”	no numerical data in the original article (values reported graphically or not reported)
Table colour coding	
Pink (light)	FWD > BL (forwards scored higher)
Blue (light)	BL > FWD (backs scored higher)
Grey	NS-no significant positional difference

## Appendix A

- Scopus

TITLE-ABS-KEY(rugby OR “rugby union” OR “rugby league” OR “rugby football”) AND TITLE-ABS-KEY(position* OR positional OR “position-specific” OR “position specific” OR forward* OR backs OR “front row” OR “back row” OR backline OR “tight five” OR “loose forward*” OR “inside back*” OR “outside back*” OR prop OR props OR hooker* OR lock OR locks OR flanker* OR “number eight” OR “scrum half” OR “fly half” OR halfback* OR centre* OR center* OR wing OR winger* OR fullback*) AND TITLE-ABS-KEY(strength OR power OR “muscular strength” OR “muscular power” OR “1RM” OR “one repetition maximum” OR “bench press” OR squat OR “power clean” OR deadlift OR isometric OR isokinetic OR “peak force” OR “rate of force development” OR “force-velocity” OR “mid-thigh pull” OR “midthigh pull” OR sprint* OR speed OR acceleration OR velocity OR “countermovement jump” OR “squat jump” OR “vertical jump” OR “jump height” OR “peak power” OR “bench throw” OR “jump squat” OR agility OR “change of direction” OR “repeated sprint” OR aerobic OR anaerobic OR “VO_2_max” OR “VO_2_” OR “VO_2_peak” OR “oxygen uptake” OR endurance OR “Yo-Yo” OR “shuttle run” OR “beep test” OR “30-15” OR “Wingate” OR “physical fitness” OR “physical performance” OR “physical characteristics” OR “physiological characteristics” OR “physical profile*” OR “physical qualities” OR “fitness testing” OR “performance testing” OR “fitness profile*”) AND NOT TITLE(“systematic review” OR “meta-analysis” OR “scoping review” OR “narrative review”) AND (LIMIT-TO (DOCTYPE,”ar”)) AND (LIMIT-TO (LANGUAGE,”English”))

- Web of Science

TS = (rugby OR “rugby union” OR “rugby league” OR “rugby football”) AND TS = (position* OR positional OR “position-specific” OR “position specific” OR forward* OR backs OR “front row” OR “back row” OR backline OR “tight five” OR “loose forward*” OR “inside back*” OR “outside back*” OR prop OR props OR hooker* OR lock OR locks OR flanker* OR “number eight” OR “scrum half” OR “fly half” OR halfback* OR centre* OR center* OR wing OR winger* OR fullback*) AND TS = (strength OR power OR “muscular strength” OR “muscular power” OR “1RM” OR “one repetition maximum” OR “bench press” OR squat OR “power clean” OR deadlift OR isometric OR isokinetic OR “peak force” OR “rate of force development” OR “force-velocity” OR “mid-thigh pull” OR “midthigh pull” OR sprint* OR speed OR acceleration OR velocity OR “countermovement jump” OR “squat jump” OR “vertical jump” OR “jump height” OR “peak power” OR “bench throw” OR “jump squat” OR agility OR “change of direction” OR “repeated sprint” OR aerobic OR anaerobic OR “VO_2_max” OR “VO_2_” OR “VO_2_peak” OR “oxygen uptake” OR endurance OR “Yo-Yo” OR “shuttle run” OR “beep test” OR “30-15” OR “Wingate” OR “physical fitness” OR “physical performance” OR “physical characteristics” OR “physiological characteristics” OR “physical profile*” OR “physical qualities” OR “fitness testing” OR “performance testing” OR “fitness profile*”) NOT TI = (“systematic review” OR “meta-analysis” OR “scoping review” OR “narrative review”)

- SPORTDiscus

(TI(rugby OR “rugby union” OR “rugby league” OR “rugby football”) OR AB(rugby OR “rugby union” OR “rugby league” OR “rugby football”) OR DE(rugby OR “rugby union” OR “rugby league” OR “rugby football”)) AND (TI(position* OR positional OR “position-specific” OR “position specific” OR forward* OR backs OR “front row” OR “back row” OR backline OR “tight five” OR “loose forward*” OR “inside back*” OR “outside back*” OR prop OR props OR hooker* OR lock OR locks OR flanker* OR “number eight” OR “scrum half” OR “fly half” OR halfback* OR centre* OR center* OR wing OR winger* OR fullback*) OR AB(position* OR positional OR “position-specific” OR “position specific” OR forward* OR backs OR “front row” OR “back row” OR backline OR “tight five” OR “loose forward*” OR “inside back*” OR “outside back*” OR prop OR props OR hooker* OR lock OR locks OR flanker* OR “number eight” OR “scrum half” OR “fly half” OR halfback* OR centre* OR center* OR wing OR winger* OR fullback*) OR DE(position* OR positional OR “position-specific” OR “position specific” OR forward* OR backs OR “front row” OR “back row”)) AND (TI(strength OR power OR “muscular strength” OR “muscular power” OR “1RM” OR “one repetition maximum” OR “bench press” OR squat OR “power clean” OR deadlift OR isometric OR isokinetic OR “peak force” OR “rate of force development” OR “force-velocity” OR “mid-thigh pull” OR sprint* OR speed OR acceleration OR velocity OR “countermovement jump” OR “squat jump” OR “vertical jump” OR “jump height” OR “peak power” OR agility OR “change of direction” OR “repeated sprint” OR aerobic OR anaerobic OR “VO_2_max” OR “VO_2_” OR endurance OR “Yo-Yo” OR “shuttle run” OR “beep test” OR “physical fitness” OR “physical performance” OR “physical characteristics” OR “physiological characteristics” OR “physical profile*” OR “physical qualities” OR “fitness profile*”) OR AB(strength OR power OR “muscular strength” OR “muscular power” OR “1RM” OR “one repetition maximum” OR “bench press” OR squat OR “power clean” OR deadlift OR isometric OR isokinetic OR “peak force” OR “rate of force development” OR “force-velocity” OR “mid-thigh pull” OR sprint* OR speed OR acceleration OR velocity OR “countermovement jump” OR “squat jump” OR “vertical jump” OR “jump height” OR “peak power” OR agility OR “change of direction” OR “repeated sprint” OR aerobic OR anaerobic OR “VO_2_max” OR “VO_2_” OR endurance OR “Yo-Yo” OR “shuttle run” OR “beep test” OR “physical fitness” OR “physical performance” OR “physical characteristics” OR “physiological characteristics” OR “physical profile*” OR “physical qualities” OR “fitness profile*”) OR DE(strength OR power OR sprint* OR speed OR agility OR aerobic OR anaerobic OR “physical fitness” OR “physical performance” OR endurance OR “vertical jump”)) NOT TI(“systematic review” OR “meta-analysis” OR “scoping review” OR “narrative review”)

- PubMed

((rugby[tiab] OR “rugby union”[tiab] OR “rugby league”[tiab] OR “rugby football”[tiab]) AND (position*[tiab] OR positional[tiab] OR “position-specific”[tiab] OR “position specific”[tiab] OR forward*[tiab] OR “backs”[tiab] OR “front row”[tiab] OR “back row”[tiab] OR backline[tiab] OR “tight five”[tiab] OR “loose forward*”[tiab] OR “inside back*”[tiab] OR “outside back*”[tiab] OR prop[tiab] OR props[tiab] OR hooker*[tiab] OR lock[tiab] OR locks[tiab] OR flanker*[tiab] OR “number eight”[tiab] OR “scrum half”[tiab] OR “fly half”[tiab] OR halfback*[tiab] OR centre*[tiab] OR center*[tiab] OR wing[tiab] OR winger*[tiab] OR fullback*[tiab]) AND (strength[tiab] OR power[tiab] OR “muscular strength”[tiab] OR “muscular power”[tiab] OR “1RM”[tiab] OR “one repetition maximum”[tiab] OR “bench press”[tiab] OR squat[tiab] OR “power clean”[tiab] OR deadlift[tiab] OR isometric[tiab] OR isokinetic[tiab] OR “peak force”[tiab] OR “rate of force development”[tiab] OR “force-velocity”[tiab] OR “mid-thigh pull”[tiab] OR “midthigh pull”[tiab] OR sprint*[tiab] OR speed[tiab] OR acceleration[tiab] OR velocity[tiab] OR “countermovement jump”[tiab] OR “squat jump”[tiab] OR “vertical jump”[tiab] OR “jump height”[tiab] OR “peak power”[tiab] OR “bench throw”[tiab] OR “jump squat”[tiab] OR agility[tiab] OR “change of direction”[tiab] OR “repeated sprint”[tiab] OR aerobic[tiab] OR anaerobic[tiab] OR “VO_2_max”[tiab] OR “VO_2_”[tiab] OR “VO_2_peak”[tiab] OR “oxygen uptake”[tiab] OR endurance[tiab] OR “Yo-Yo”[tiab] OR “shuttle run”[tiab] OR “beep test”[tiab] OR “30-15”[tiab] OR “Wingate”[tiab] OR “physical fitness”[tiab] OR “physical performance”[tiab] OR “physical characteristics”[tiab] OR “physiological characteristics”[tiab] OR “physical profile*”[tiab] OR “physical qualities”[tiab] OR “fitness testing”[tiab] OR “performance testing”[tiab] OR “fitness profile*”[tiab])) NOT (“systematic review”[ti] OR “meta-analysis”[ti] OR “scoping review”[ti] OR “narrative review”[ti])
